# How study-related positive emotions and academic psychological capital mediate between teacher-student relationship and academic performance: a four-wave study among high school students

**DOI:** 10.3389/fpsyg.2024.1419045

**Published:** 2024-08-29

**Authors:** Marcos Carmona-Halty, Karina Alarcón-Castillo, Carla Semir-González, Geraldy Sepúlveda-Páez, Patricio Mena-Chamorro, Francisca Barrueto-Opazo, Marisa Salanova

**Affiliations:** ^1^Escuela de Psicología y Filosofía, Universidad de Tarapacá, Arica, Chile; ^2^Escuela de Sociología, Universidad Católica del Maule, Talca, Chile; ^3^WANT Research Team, Universitat Jaume I, Castellón de la Plana, Spain

**Keywords:** teacher-student relationship, study-related positive emotions, academic PsyCap, academic performance, high school students

## Abstract

This article presents a theory-driven model in which teacher-student relationships and academic performance are indirectly related through study-related positive emotions and academic psychological capital. A sample of 1,054 Chilean high school students (50.4% females) aged 12–17 (*M* = 14.46, SD = 1.74) participated in the study. Through structural equation modeling, the direct and indirect effects of the proposed model were calculated. The results show that study-related positive emotions and academic psychological capital mediate between the teacher-student relationship and academic performance. These results have significant implications for improving teaching competencies through positive psychological interventions aimed at developing skills in students and thus improving students' academic performance and general well-being in educational settings.

## Introduction

There has been an emergence in recent years of positive education as a growing body of research that contributes to promoting students' mental health and optimal functioning (Seligman et al., 2009; Seligman and Adler, 2019; Tejada-Gallardo et al., 2020; Goetz et al., 2021). Considerable attention has been directed toward the impact of affective qualities within teacher-student relationships on fostering academic engagement (Roorda et al., 2011, 2017, 2019). However, little is known about other constructs' role in the association between teacher-student relationships and academic performance (King, 2015; Roorda et al., 2017; Carmona-Halty et al., 2019b). This report aims to fill this gap by presenting a theory-driven model, drawing upon the self-determination (SD) theory (Ryan and Deci, 2000), the broaden-and-build (B&B) theory (Fredrickson, 1998), and the conservation of resources (COR) theory (Hobfoll, 1989). The model explores how study-related positive emotions (SPE) and academic psychological capital (APC) mediate the relationship between teacher-student relationship (TSR) and academic performance (AP). In other words, the study aimed to examine the indirect relationships between TSR and AP through sequential mediation by SPE and APC. The validation of the proposed model could have implications for implementing future evidence-based interventions to enhance student wellbeing and academic performance, aligning with the objectives of positive education.

Teachers represent one of the most important social relationships for young people and can be seen as psychological parents who provide a safe haven for their pupils (Fabris et al., 2022). SD theory asserts that the basic human needs for relatedness, competence, and autonomy form the basis for the development and motivation of an individual's behavior (Ryan and Deci, 2000). In synthesis, *autonomy* refers to feeling willingness and volition regarding one's behaviors; *competence* refers to feeling effective in one's interactions with the social environment; and *relatedness* refers to experiencing that others are responsive and sensitive, as well as being able to be responsive and sensitive to others (Ryan and Deci, 2000). Teachers who are successful in satisfying these needs promote student engagement, wellbeing, and academic achievement (Howard et al., 2024). Hence, teachers can satisfy their students' basic psychological needs for relatedness, provide emotional support, and make them feel safe to explore their environment and cope with academic demands (Ryan and Deci, 2000). Along this line, previous research has shown that the relationship between teacher and student is directly related to engagement (King, 2015), personal resources (Carmona-Halty et al., 2019b), learning (Li and Zhang, 2024), well-being (Lin et al., 2021), motivation (Wang et al., 2024), and performance (Hajovsky et al., 2017).

Positive emotions have proven to be a relevant antecedent in the learning processes, performance, and students' wellbeing (e.g., Pekrun and Linnenbrink-Garcia, 2012; Carmona-Halty et al., 2019a; Lee et al., 2021). They arise when people perceive good prospects or good fortune (Fredrickson, 2013). According to the B&B theory (Fredrickson, 1998)—which specifies the “broaden hypothesis” and the “build hypothesis”—experiencing positive emotions increases people's responses and solutions to everyday challenges (i.e., *broaden*); therefore, it is possible to discover and devise new and more durable personal resources is made possible (i.e., *build*; Fredrickson, 2004). Therefore, the range of people's thoughts, actions, skills and personal resources is expanded (Fredrickson, 2013). Thus, it can be said that students who experience positive emotions expand their cognitive and behavioral repertoire in the face of daily challenges. Along this line, previous research has provided substantial evidence that SPE are associated to students' autonomy (Meuleners et al., 2022), learning satisfaction (Lee et al., 2021), cognitive appraisals integration (Forsblom et al., 2022), personal resources (Ouweneel et al., 2011), engagement (Oriol-Granado et al., 2017), and performance (Carmona-Halty et al., 2019a).

Personal resources, defined as characteristics that are inherently valued or serve as means to attain or safeguard valued resources (Diener and Fujita, 1995), are significant predictors of wellbeing and performance (Dogan, 2015; Rand et al., 2020; Etherton et al., 2022). According to the COR theory, resources are not isolated entities; individuals, as a means of adapting to their environment, endeavor to accumulate resources within resource caravans (Hobfoll, 1989, 2002). Following this trend, an example of resource caravans (i.e., a combination of personal resources) is the APC construct, a developmental psychological state composed of the personal resources of hope, efficacy, resilience, and optimism. Hope refers to persevering with goals and, when necessary, redirecting paths toward goals to be successful. Efficacy is concerned with having the confidence to accept and exert the effort necessary to accomplish challenging tasks. Resilience is about endurance, recovery, and even going beyond to achieve success when faced with problems and adversity (Luthans and Youssef-Morgan, 2017). Research has shown that academic PsyCap is associated with motivation (Datu et al., 2016), engagement (Datu and Valdez, 2016), coping (Ramírez-Pérez, 2022), life satisfaction (Xu and Choi, 2023), well-being (Finch et al., 2023), and performance (Sánchez-Cardona et al., 2021).

The constructs mentioned and the research carried out allow us to hypothesize a sequential model based on the theories underpinning them. More specifically, the present study proposes a theory-driven model that examines how SPE and APC mediate between TSR and AP. The reasoning behind the proposed model is as follows: if students have a relationship with their teachers that satisfies their basic psychological needs, they are more likely to experience SPE, which would enhance personal resources, such as APC; and, consequently, a better AP will be achieved. Additionally, considering previous research on the role of gender between TSR and AP, the gender invariance of the proposed model will be assessed (e.g., Hajovsky et al., 2017; Goldie and O'Connor, 2021).

## Materials and methods

### Participants

The sample included 1,054 (50.4% females) high school students aged 12–17 years (*M* = 14.46, SD = 1.74) from three different schools in the Tarapacá region of northern Chile (each school hosted ~700 students). Of the 1,054 students, 18% were 12 years old, 17% were 13 years old, 20% were 14 years old, 21% were 15 years old, 13% were 16 years old, and 11% were 17 years old at the time of data collection.

### Measures

At time 1, the TSR was measured using an adapted version of the *Teacher-Student Relationship Scale* developed by Martin et al. (2007), adapted to Spanish and applied in Chilean population by Carmona-Halty et al. (2019b). The scale has four items (e.g., “*My teachers give me the help and support I need*”) and is scored on a 7-point rating scale from 1 (strongly disagree) to 7 (strongly agree). At time 2, after 9 weeks SPE was measured using six items—scored on a scale of 1 (*never*) to 5 (*always*)—from the *Job-related Affective Well-being Scale* (Van Katwyk et al., 2000), adapted to the academic context (e.g., “*My studies make me feel at ease*”) and applied to the Chilean population by Carmona-Halty et al. (2019a). At time 3, after another 9 weeks, APC was measured using a short form of the *Academic Psychological Capital Questionnaire*, adapted to Spanish and applied to the Chilean population by Martínez et al. (2021). This questionnaire has five items that measure hope, efficacy, resilience, and optimism (e.g., “*I usually take stressful things in stride about my studies*”) on a 6-point rating scale from 1 (*strongly disagree*) to 6 (*strongly agree*). At time 4, following an additional 9-week period, AP was assessed using the grade point average (GPA) obtained from educational institutions. The assessment considered four compulsory subjects in the Chilean educational curriculum: mathematics, language, history, and science, graded on a scale from 1 (poor) to 7 (excellent).

### Procedure

This study was conducted as part of a project to investigate the influence of non-intellectual variables on academic performance. Students voluntarily completed a questionnaire at three points during the academic year with a time interval of 9 weeks between each. Additionally, at the end of the academic year, the GPA was obtained from the class books 9 weeks later. Data collection was carried out in the form of online questionnaire sessions. Participants spent between 5 and 10 min each time answering the questionnaire and were always given the same instructions.

### Data analysis

First, means, standard deviations, Cronbach's alpha and McDonald's omega reliability indices, and Pearson's correlation coefficient were calculated using the statistical analysis software Jamovi version 1.8.1 (The Jamovi Project, 2020). Second, a structural equation model (SEM) was estimated to examine the effect of the TSR on AP through SPE and APC, using Mplus v8.2 (Muthén and Muthén, 1998–2017). For this purpose, the Weighted Least Square mean and Variance adjusted (WLSMV), which is robust with discrete variables without normal distribution, were considered (Liang and Yang, 2014; Li, 2016). The goodness-of-fit was assessed according to the guidelines proposed by Hair et al. (2019) for models with sample sizes larger than 250 participants and with more than 12 observed variables. Third, direct and indirect effects were examined by implementing the bootstrap procedure with 5,000 re-samples, constructing 95% bias-corrected and accelerated (BCa) confidence intervals (CI). Fourth, gender invariance was examined through multiple-group SEM, and three levels of equivalence were assessed using changes in CFI and RMSEA as criteria for determining whether measurement invariance was established (Cheung and Rensvold, 2002; Chen, 2007).

## Results

Table 1 shows means, standard deviations, Cronbach's and McDonald's indexes, and Pearson's correlations among the variables. The internal consistencies of the scales, as indicated by Cronbach's alpha (ranging from 0.875 to 0.922) and McDonald's omega (ranging from 0.885 to 0.923), were deemed satisfactory. Moreover, the pattern of correlations revealed significant direct associations across all criteria.

**Table 1:** Descriptive statistics, reliability indices and Pearson correlation matrix (*n* = 1,054).

	***M* (SD)**	**α**	**ω**	**TSR**	**SPE**	**APC**	**AP**
Teacher-student relationship (TSR)	5.163 (1.390)	0.890	0.891	–			
Study-related positive emotions (SPE)	2.984 (0.945)	0.922	0.923	0.345^*^	–		
Academic PsyCap (APC)	4.361 (1.101)	0.884	0.885	0.402^*^	0.541^*^	–	
Academic performance (AP)	5.382 (0.757)	0.875	0.875	0.120^*^	0.173^*^	306^*^	–

^*^p < 0.001.

**Figure 1:**
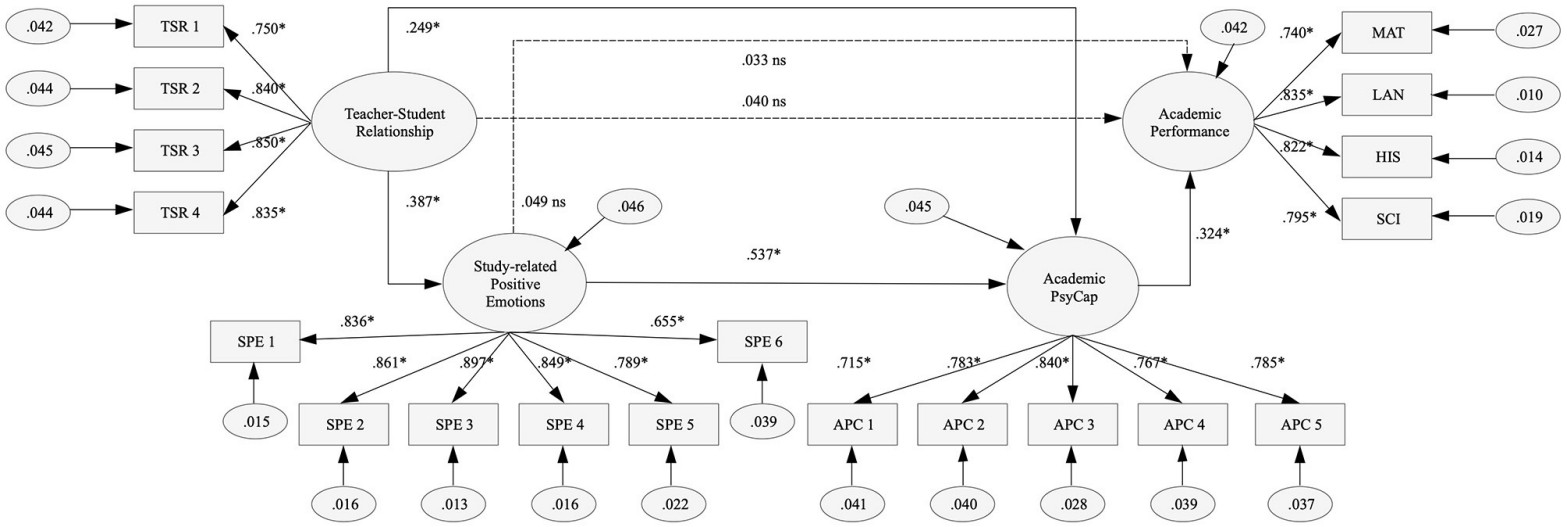
Graphical representation of the proposed theory-driven model with standardized coefficients, *p < 0.001; ns, non-significant effect.

The hypothesized model (Figure 1) adequately fits the data (see Table 2, M1), explaining 15.0, 45.3, and 10.8% of the variance of SPE, APC, and AP, respectively. Considering the adequate fit of the model, direct and indirect effects were calculated. First, we observed direct and significant effects between TSR and SPE [β = 0.387, 95% CI (0.317, 0.456), *SE* = 0.035, *p* < 0.001], and TSR and APC [β = 0.249, 95% CI (0.181, 0.316), *SE* = 0.034, *p* < 0.001]; between SPE and APC [β = 0.537, 95% CI (0.467, 0.600), *SE* = 0.034, *p* < 0.001[; and APC and AP [β = 0.324, 95% CI (0.226, 0.417), *SE* = 0.049, *p* < 0.001]. Second, we observed an indirect effect between TSR and AP through SPE and APC [β = 0.160, 95% CI (0.120, 0.209), *SE* = 0.022, *p* < 0.001]. The multiple-group SEM shows that the differences in the CFI across the three invariance models were lower than 0.01, which indicated gender invariance (see Table 2).

**Table 2:** Fit indexes for single–group and multiple–group CFA of the theory-driven model.

	**χ^2^**	***df***	**χ^2^/*df***	**RMSEA**	**90% CI**	**CFI**	**TLI**	**SRMR**	**CMs**	**Δ CFI**
**Single–group CFA**										
M1 theory-driven model	535.441^*^	*146*	3.667	0.050	(0.046, 0.055)	0.969	0.964	0.042	–	–
**Multiple–group CFA**										
M2 configural invariance	718.811^*^	292	2.462	0.037	(0.034, 0.041)	0.966	0.961	0.043	–	–
M3 metric invariance	736.125^*^	307	2.398	0.036	(0.036, 0.033)	0.966	0.962	0.049	M2–M3	0.000
M4 scalar invariance	848.939^*^	326	2.604	0.039	(0.039, 0.036)	0.965	0.957	0.049	M3–M4	0.001

^*^p < 0.001.

χ2, chi-square; *df*, degree of freedom; RMSEA, Root Mean Square Error of Approximation; CI, 90% confidence interval; CFI, Comparative Fit Index; TLI, Tucker-Lewis Index; SRMSR, Standardized Root Mean Square Residual; CMs, comparisons between models.

## Discussion

This brief report makes an innovative contribution by proposing and empirically validating a theory-driven model that integrates relevant developments for the discipline. Below are discussed the findings' theoretical and practical implications and a breakdown of the study's strengths and weaknesses, followed by considerations for future research directions.

Consistent with prior research in SD theory, our study indicated that students who perceive positive relationships with their teachers, characterized by feeling listened to, valued, and supported, are more likely to experience positive emotions such as relaxation, enthusiasm, inspiration, and satisfaction concerning their studies (e.g., Roorda et al., 2017; Carmona-Halty et al., 2019c; Lin et al., 2021). In a similar vein, consistent with previous research on B&B theory, when students experience positive emotions about their studies, they are more likely to accumulate the personal resources (in the form of hope, efficacy, resilience, and optimism) necessary to deal with their academic demands (Ouweneel et al., 2011; Oriol-Granado et al., 2017; Carmona-Halty et al., 2019a). Finally, consistent with previous research on COR theory, students who manage to accumulate enough personal resources are more likely to deploy these resources to achieve adequate academic performance (Datu et al., 2016; Ortega-Maldonado and Salanova, 2018; Carmona-Halty et al., 2019b). Taken together, our study makes an innovative contribution to research on the association between TSR and AP by emphasizing the intermediate role of SPE and APC.

Based on the SD theory, educational institutions could train their teachers to promote high-quality of TSR by focusing on creating a friendly, affectionate, and supportive classroom atmosphere. In this atmosphere, teachers should be able to get to know their students, dedicate time, listen, and provide support (Kincade et al., 2020). In addition, drawing on the B&B theory, teachers could foster a classroom climate that promotes this experience. This experience can be achieved by positively reinforcing effective classroom instruction, encouraging kind and caring words, fostering collaborative work among peers, and incorporating educational games that contribute to learning (Li et al., 2020). Finally, drawing on COR theory, developing an APC, a training model for a PsyCap intervention, could be promoted (Luthans et al., 2013; Finch et al., 2023). Taken together, our results show that it could be beneficial to implement positive psychological interventions (PPIs) in schools (Shankland and Rosset, 2017). However, these proposals should consider the health status of teachers, and not imply an additional workload, considering that their well-being predicts their students' performance (Hascher and Waber, 2021).

The strengths of the current study are the longitudinal approach and the sample size; the inclusion of an objective measure of achievement; and the successfully integrated SD, B&B, and COR theories, which, as far as we know, have never been tested previously. However, the study has several limitations. First, our data sample does not represent the entire population of Chilean students; therefore, caution is advised when generalizing the results. Second, our analysis only considers unidirectional effects rather than estimating bidirectional effects (including autoregressive and reciprocal effects). Third, our study's four assessment points are limited to capturing short-term effects rather than exploring long-term effects. Finally, as considerations for future research can be mentioned first, incorporate the role of additional significant others such as parents or classmates, which has also been shown to predict academic wellbeing and performance; second, a group-level of the variables considered in our model (i.e., teacher-class relationship, class study-related positive emotions, class academic PsyCap), further exploration could be conducted to explore its role in students' academic performance. Additionally, other facets of the teacher-student relationship could be considered such as conflict or dependency.

## Data availability statement

The raw data supporting the conclusions of this article will be made available by the authors, without undue reservation.

## Ethics statement

The studies involving humans were approved by Comité Ético-Científico/Universidad de Tarapacá (CEC-UTA). The studies were conducted in accordance with the local legislation and institutional requirements. Written informed consent for participation in this study was provided by the participants' legal guardians/next of kin.

## Author contributions

MC-H: Writing – original draft, Writing – review & editing, Conceptualization, Methodology, Investigation. KA-C: Writing – original draft, Writing – review & editing. CS-G: Writing – original draft, Writing – review & editing. GS-P: Writing – original draft, Writing – review & editing. PM-C: Writing – review & editing, Writing – original draft. FB-O: Writing – review & editing, Writing – original draft. MS: Writing – original draft, Writing – review & editing.
